# An MRI Study of Symptomatic Adhesive Capsulitis

**DOI:** 10.1371/journal.pone.0047277

**Published:** 2012-10-17

**Authors:** Wen Zhao, Xiaofeng Zheng, Yuying Liu, Wenlu Yang, Vardan Amirbekian, Luis E. Diaz, Xudong Huang

**Affiliations:** 1 Department of Orthopedics, Beijing Aerospace General Hospital, Beijing, China; 2 Department of Radiology, Beijing Aerospace General Hospital, Beijing, China; 3 Department of Cell and Molecular Pharmacology and Experimental Therapeutics, Medical University of South Carolina, Charleston, South Carolina, United States of America; 4 Department of Electrical Engineering, Information Engineering College, Shanghai Maritime University, Shanghai, China; 5 Department of Radiology, Brigham and Women's Hospital and Harvard Medical School, Boston, Massachusetts, United States of America; 6 Department of Radiology, Boston University School of Medicine and VA Boston Healthcare System, Boston, Massachusetts, United States of America; 7 Department of Psychiatry, Massachusetts General Hospital and Harvard Medical School, Charlestown, Massachusetts, United States of America; University of California, United States of America

## Abstract

**Background:**

Appilication of MR imaging to diagnose Adhesive Capsulitis (AC) has previously been described. However, there is insufficient information available for the MRI analysis of AC. This study is to describe and evaluate the pathomorphology of the shoulder in Asian patients with AC compared to healthy volunteers.

**Methodology/Principal Findings:**

60 Asian patients with clinically diagnosed AC and 60 healthy volunteers without frozen shoulder underwent MRI of the shoulder joint. All subjects who were age- and sex-matched control ones underwent routine MRI scans of the affected shoulder, including axial, oblique coronal, oblique sagittal T1WI SE and coronal oblique T2WI FSE sequences. Significant abnormal findings were observed on MRI, especially at the rotator cuff interval. The coracohumeral ligament (CHL), articular capsule thickness in the rotator cuff interval as well as the fat space under coracoid process were evaluated. MRI showed that patients with adhesive capsulitis had a significantly thickened coracohumeral ligament and articular capsule in the rotator cuff interval compared to the control subjects (4.2 vs. 2.4 mm, 7.2 vs. 4.4 mm; p<0.05). Partial or complete obliteration of the subcoracoid fat triangle was significantly more frequent in patients with adhesive capsulitis compared with control subjects (73% vs. 13%, 26% vs. 1.6%; p<0.001). Synovitis-like abnormality around the long biceps tendon was significantly more common in patients with adhesive capsulitis than in control subjects. With regards to the inter-observer variability, two MR radiologists had an excellent kappa value of 0.86.

**Conclusions/Significance:**

MRI can be used to show characteristic findings in diagnosing AC. Thickening of the CHL and the capsule at the rotator cuff interval and complete obliteration of the fat triangle under the coracoid process have been shown to be the most characteristic MR findings seen with AC.

## Introduction

Adhesive capsulitis (AC) is also known as scapulohumeral periarthritis or “frozen shoulder,” a term coined in 1934 by Codman [1]. AC is a diffuse inflammatory process involving all of the soft tissue components of the scapulohumeral joint including the synovial surface of the joint capsule, the glenohumeral ligaments, the periarticular tendons and bursae (particularly the subacromial bursae), or the biceps tendon sheath. A concomitant finding in all cases of frozen shoulder, either primary or secondary in its origin, is biceps tendon tenosynovitis, which is thought to be the cause of pain and the most distressing feature of this syndrome [2]. Clinical symptoms of AC include a gradual onset of pain, inability to sleep on the affected arm, restriction of both active and passive elevation as well as restricted external rotation. AC has either primary/idiopathic causes or occurs as sequelae of rheumatoid arthritis, osteoarthritis, fracture, or joint dislocation. The estimated prevalence of AC is 2% to 3% in the general population [3] and 5% to 6% in patients evaluated by shoulder surgeons [4].

Imaging can assist in the diagnosis of AC with certain degrees of accuracy, depending on the modality used. Adjunct imaging, mainly radiographs, shows shoulder osteoporosis, subacromial calcification, or increase density and shallowing of the intertubercular groove of the humerus. However, radiographs are difficult to use of accurate and specific assessment as the results are usually negative and only serve to exclude other causes of shoulder pain. Attempts have been made to accurately diagnose AC through imaging modalities including MRI [5], [6], ultrasound [7], nuclear medicine [8], and arthrography [9]. MRI is generally regarded as the gold standard for shoulder imaging because of its superior contrast resolution, optimal soft tissue visualization, multi-planar scanning capabilities, as well as non-invasive nature [10]. More recently, MRI features can enhance the diagnostic accuracy of AC in the literature [5], [6], [11], [12]. However, these results were confusing. For example, Eming et al found that there was no significant difference on the thickness of the coracohumeral ligament between patients with AC and asymptomatic volunteers, confirming the rotator cuff interval was not useful for assessing changes of adhesive capsulities [13]. Similar result reported by Manton et al who found there were no significant differences in 28 patients on the capsular and synovial thickness with MRI arthrography between symptomatic and asymptomatic group [14]. However, Mengiradi et al reported that the thickening of the CHL and the joint capsule in the rotator cuff interval, as well as the subcoracoid triangle sign, are characteristic MR arthrographic findings in frozen shoulder [15].

Accordingly, understanding the pathomorphology of AC is important in its diagnosis and treatment, MR imaging may provide an important additional diagnostic tool for AC disease. Therefore we quantitatively evaluate the MR imaging pattern in Asian patients with AC.

## Methods

### Objectives

In this study, we want to determine whether structurally pathological changes of the rotator cuff interval detected by MRI can be used for the MRI diagnosis of adhesive capsulitis. We will further confirm previous characteristic MR arthrographic findings in frozen shoulder [6], [15].

### Participants

60 shoulder joints in 60 Asian patients were evaluated by two experienced shoulder surgeons between July 2006 and June 2009. Patients were eligible for this study if they were clinically diagnosed with AC. All patients reported an insidious onset of shoulder pain and dysfunction with a time period ranging from 15 weeks to 30 months (mean 12 months). Clinical criteria for diagnosis of AC included pain and stiffness for greater than fifteen weeks that was increasing in intensity, was at its most severe at rest, with restriction of passive motion greater than 30° in two or more planes of movement. Exclusion criteria included previous shoulder surgery, history of shoulder trauma, neurological disorders involving the upper limbs, clinical history and clinical examination compatible with rotator cuff tear, presence of calcium deposition on radiographic evaluation, rheumatoid arthritis or osteoarthritis.

Mean age of patients was 50.2 years (age range 36–74 years). There were 36 women and 24 men in this study. Sixty healthy asymptomatic volunteers (24 men, 36 women; mean age 46.9 years) served as a control group and underwent shoulder MRI using the same protocol as the symptomatic group. The experiments were performed and all data were collected at the Beijing Aerospace General Hospital.

### MR Procedures and Image Analysis

Patients were examined with a 0.5-T superconducting unit (Signa Contour, GE Medical Systems, Milwaukee, WI, USA) at two clinical sites. The shoulder was scanned in neutral position with an upward thumb or natural position with external rotation that avoids an overlap of the supraspinatus and infraspinatus tendons at the oblique coronal plane. The patients were in supine position with their arms placed in neutral position by their side. A surface coil (Med Rad multipurpose array, Indianola, PA, USA) was used centered over the glenohumeral joint and strapped in place.

Spin-echo (SE) T1W fat-saturated sequences were obtained in the coronal oblique, sagittal oblique, and axial planes. Axial sequences were performed with TR/TE 300/17 ms, slice thickness 4 mm with an interslice gap of 1 mm, field of view (FOV) 14–20 cm, matrix 160×256, 3 number of excitations. The scanning range for the axial plane covered from the acromioclavicular joint to the inferior plane of the glenoid cavity. Sagittal oblique and coronal oblique T1W images were obtained with TR/TE 660/20, slice thickness 4 mm with a gap of 1 mm, matrix 160×256, NEX 3, and FOV 14–20 cm. The scanning range for the oblique sagittal plane covers the glenoid cavity to the outermost aspect of the head of humerus, which was parallel with the plane of the glenoid or perpendicular with the supraspinatus tendon; for oblique coronal plane, the range covered from the subscapularis tendon from front to rear, which was perpendicular with the plane of the glenoid or parallel with the tendon of the supraspinatus muscle. The SE T2WI fat-saturated sequences were obtained in the coronal oblique plane. The scanning area of the SE T2WI was the same as the SE T1WI sequence. The coronal oblique T2WI images were obtained with TR/TE 2880/70, slice thickness 4 mm with a gap of 1 mm, matrix 160×256, FOV 14–20 cm and ETL 8. The STIR fat suppressed images were obtained with TR/TE 3800/25, slice thickness 4 mm with a gap of 1 mm, matrix 256×160, FOV 24 cm, bandwidth 10.42 and ETL 8.

Two experienced (more than 5 years) MR radiologists, who were blinded to the patient's clinical stage of AC, read the MRI examinations by means of consensus. The inter-observer variability between these two radiologists was determined by using a SPSS 13.0 statistical software package. They had an excellent kappa value of 0.86. The interpretation represents the original report. The specific MRI findings were evaluated using established techniques [15]. In brief, the thickness of the coracohumeral ligament was measured on the sagittal oblique images and the thickness of the capsule was determined at the rotator cuff interval. The rotator cuff interval was identified by the previouly defined boundaries and assessed for the presence of increased signal intensity [13]. Partial or complete obliteration of the subcoracoid fat triangle was determined in the sagittal oblique plane. Borders of the triangle were defined anterosuperiorly by the coracoid process and coracobrachialis, superiorly by the CHL, and posteroinferiorly by the joint capsule. Partial disappearance of the fat signal was considered partial obliteration of the subcoracoid fat triangle under the coracoid process, and complete disappearance of fat signal was considered complete obliteration. Other criteria were evaluated and characterized as present or absent, such as synovitis-like abnormality at the superior border of the subscapularis tendon, around the long biceps tendon, and around the supraspinatus muscle tendon. Synovitis-like abnormalities were diagnosed on the basis of evidence of synovial irregularity and/or thickening. The articular surface of the subscapularis tendon was shown as a high signal intensity structure on the sagittal oblique T2-weighted images.

**Figure 1:**
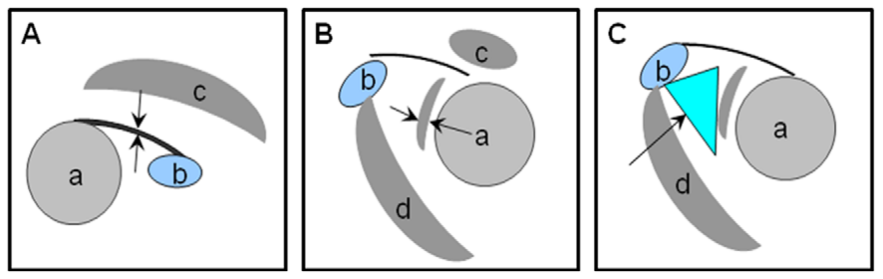
Measurement Scheme. (**A**) Thickest portion of CHL on sagittal oblique T1-weighted spin-echo images was measured (arrowheads). a, caput humeri, b, coracoid notch, c, supraspinatus muscle. (**B**) Capsular thickness (arrow) in rotator cuff interval was measured on sagittal oblique T1-weighted spin-echo images. Thickest portion of capsule (arrow) perpendicular to surface of humeral head was measured along a radial line drawn to center of humeral head. a, caput humeri, b, coracoid notch, c, supraspinatus muscle, d, coracobrachial muscle. (**C**) Sagittal oblique T1-weighted image of subcoracoid fat triangle. Borders of the triangle (arrow) are defined anterosuperiorly by the coracoid process (C), superiorly by the CHL (arrow), and posteroinferiorly by the joint capsule. a, caput humeri, b, coracoid notch, d, coracobrachial muscle.

Our MR measurement scheme is similar to the one reported by Mengiardi et al [15], and it was summarized in Figure 1.

### Ethics

Written forms of informed consent were obtained from all patients. All patients consented to have their medical records reviewed for the purposes of this study. This study was approved by the Institutional Review Board and Ethics Committee of the Beijing Aerospace General Hospital.

### Statistical Methods

Quantitative data were analyzed using standard statistical methods, ANOVA and paired student *t* tests, followed by Fisher's PLSD and Bonferroni post hoc tests. Qualitative data were compared by using the χ^2^ test. Group data are expressed as means ± SD. Values of all of the parameters were considered significantly different at a value of *p*<0.05.

## Results

**Figure 2:**
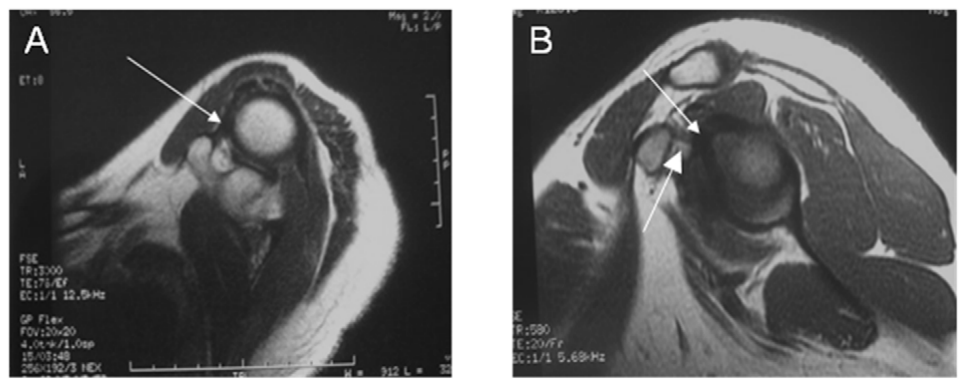
MR image shows thickened and slightly twisted CHL (arrow) in a 52-year-old patient with AC in axial plane T1WI (A) and in sagittal oblique plane (B).

The rotator cuff interval was easily identified using MR imaging in clinical patients with AC and in asymptomatic volunteers. The data were reviewed with respect to assigning a thickness value for capsule and synovium most sensitive and specific for AC. In the control group, the thickness of CHL was 2.12±0.84 mm and range from 1.0 mm to 3.5 mm. In contrast, patients with AC had a significantly thicker CHL than did control subjects (mean, 4.2 vs. 2.4 mm; range, 3.5–6.2 mm vs. 1.0–3.5 mm; p<0.001) (**Table 1** **,** **Figure 2**). Consistent with this result, Bernard et al also found that there was significant difference in the thickness of CHL between patients with AC and the control subjects (4.1 mm vs 2.7 mm in controls) [15]. They also found that the specificity of a CHL diameter of 4 mm or greater indicating the diagnosis was 95%. Thickening of the CHL is therefore one of the characteristic MRI findings in AC. Similarly, the mean thickness of the articular capsule at the rotator cuff interval was also higher in patients with AC than in control subjects (mean, 7.2 vs. 4.4 mm; range, 4.5–10 mm vs. 3.0–7.1 mm), with significant difference (p<0.05), indicating that thickening of the articular capsule can also be a useful MRI sign of AC.

**Table 1:** Thickness of CHL and articular capsule at the rotator cuff interval (60 shoulders with AC compared with 60 healthy shoulders).

	Shoulders with AC (mm)	Control group shoulders (mm)	*p* value
Thickness of CHL	4.21±0.97	2.12±0.84	0.000 (<0.001)
Thickness of articular capsule	7.20±2.13	4.43±1.16	0.027 (<0.05)

**Figure 3:**
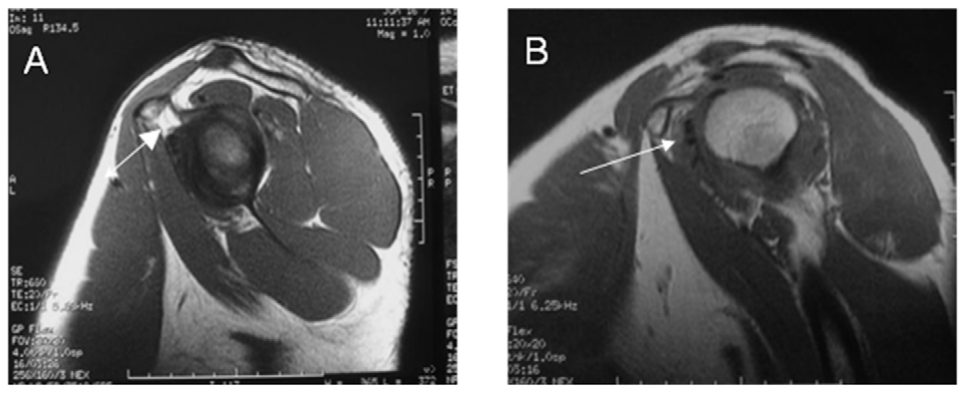
Sagittal oblique TIWI images of subcoracoid fat triangle in control subject and a 55-year-old patient with AC. (**A**) Normal subcoracoid fat triangle in control subject; (**B**) Partial obliteration of subcoracoid fat triangle.

**Figure 4:**
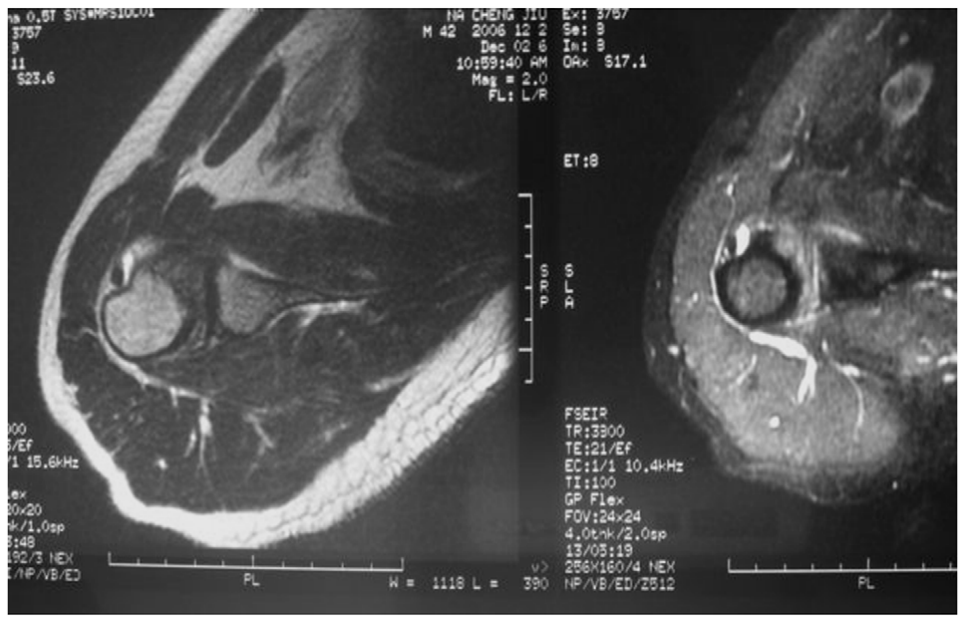
Synovitis-like abnormalities around the long biceps tendon in a 57-year-old patient of with AC. T2 prolongation (hyperintensitiy) was observed around the tendon (low signal structure).

Partial or complete obliteration of the subcoracoid fat triangle (“subcoracoid triangle sign”) was significantly more frequent in patients with AC compared with control subjects (partial obliteration, 22 vs. 2 cases (73% vs. 13%); complete obliteration, 8 vs. 1 cases (26% vs. 1.6%; p<0.001) (**Figure 3** **,** **Table 2**). Therefore, the subcoracoid triangle sign can be a characteristic finding in AC. Synovitis-like abnormalities around the long biceps tendon were also markedly more frequent in patients than in control subjects (18 vs. 2 cases (60% vs. 6%), p<0.05) (**Figure 4** **,** **Table 2**). However, patients were not significantly different from control subjects with regard to synovitis-like abnormalities at the articular surface of the subscapularis tendon or in the supraspinatus muscle tendon (46% vs. 20%; 20% vs. 0%). Our results showed that the identified measurement on MR imaging can suggest the diagnosis of AC with reasonable sensitivity and excellent specificity.

**Table 2:** Measurement parameters at shoulder joint.

	Shoulders with AC Cases (frequency)	Healthy shoulders Cases (frequency)	*p* value
Obliteration of subcoracoid fat triangle:			
Partial	22 (73%)	2 (13%)	
Complete	8 (26%)	1 (0%)	
Synovitis-like abnormalities:			
(1) Around long biceps tendon	18 (60%)	2 (6%)	0.007 (<0.01)
(2) At the subscapularis tendon	14 (46%)	6 (20%)	0.245 (>0.05)
(3) In supraspinatus muscle tendon	6 (20%)	0 (0%)	0.224 (>0.05)

## Discussion

This study confirms that MRI can be used to show specific abnormalities in patients with AC compared to healthy control subjects [6], [15]. The specific abnormalities that were associated with AC include: (i) thickening of the CHL; (ii) thickening of the rotator interval; (iii) marked obliteration of subcoracoid fat triangle; and (iv) synovitis-like abnormalities around the long biceps tendon.

AC, a clinical condition with painful restriction of shoulder movement, is a common clinical problem that causes major functional morbidity and pain, more commonly in women over 40 years of age [16], [17]. The natural history of AC generally follows two courses. Some patients respond to conservative measures such as medications, local injections, and physical therapy. Other patients cannot tolerate the pain or the restrictions on motion and elect surgical intervention. Although there is no agreement regarding the etiology of AC, there is agreement about the observed pathologic changes: inflammation combined with a fibrotic reaction leading to thickening, contraction and subsequent adherence of the capsule, synovium and surrounding ligamentous structures [13], [16], [18]. The initial stages of AC have a predominance of pain, with gradually increasing joint stiffness brought on by ongoing synovial inflammation and capsular fibrosis. In the later stages, as the inflammatory phase passes, capsular fibrosis becomes the predominant pathologic finding [6], [18]. Histologic and immunocytochemical studies have shown active fibroblastic proliferation accompanied by transformation of fibroblasts to myofibroblasts, a situation thought to initiate contracture of the coracohumeral ligament component of the rotator interval in the early stages of idiopathic AC [19]. As the rotator interval shortens in the mediolateral and craniocaudal directions, the relative motion of the anterior margin of the supraspinatus tendon and the cranial margin of the subscapularis tendon becomes restricted and external rotation range diminishes.

It has been suggested that the CHL and rotator interval are of central importance in the development of AC [20]. The CHL at the rotator interval is one of the primary structures restraining external rotation, which has particular clinical significance in AC [21], [22], [23], [24]. Indeed, Mengiardi et al found that a coracohumeral ligament thickness greater than 4 mm on sagittal oblique MR arthrographic images was associated with AC in 142 cases [15]. Consistent with this result, the CHL in the rotator cuff interval was significantly thickened in our patients with AC. However, in patients with recalcitrant chronic AC, the contracted CHL was shown to be the primary lesion and, furthermore, release of the contracted structures relieved pain and restored motion at the shoulder [25]. Omari et al also described that the CHL was transformed into a tough contracted band in 25 patients with primary AC and that the histology of these contractures consisted of a dense matrix of type III collagen populated with fibroblasts and myofibroblasts [26]. When the contracture of CHL in patients was excised, pain subsided and shoulder function was restored. Taken together, the pathologic changes observed at the CHL by may be helpful for diagnosing AC by MRI.

There is evidence that the rotator interval is important for detecting and quantitatively assessing AC [27]. The rotator interval is defined as a triangular structure with the base of the triangle as the coracoid process, the apex as the intertubercular groove, the inferior border as the superior aspect of the subscapularis tendon, and the superior border as the anterior aspect of the supraspinatus tendon. This triangular space contains the biceps tendon, superior glenohumeral ligament, the glenohumeral capsule, and the CHL [20]. In addition to thickening of the CHL on MRI, we also found that the rotator interval capsule was thickened in AC compared with control subjects. MRI investigations of AC have previously shown thickening of the rotator interval capsule and exuberant synovitis surrounding the coracohumeral ligament, which may enhance after intravenous gadolinium injection [5], [28]. Lee et al also found that with AC there was significant thickening of the joint capsule at the axillary recess using MR arthrography [29]. The results of our investigation agree with those of Carrillon, Lefecre-Colau and others, who found thickening of the joint capsule and synovial membrane at the rotator interval to be helpful in diagnosing AC [12], [30]. Moreover, in 17 of 22 patients, Mengiardi et al found an inflammatory obliteration of the subcoracoid fat triangle with AC [15], which we also observed in partial or complete form in this investigation. The complete obliteration of this fat triangle was specific to the diagnosis of frozen shoulder or AC. This subcoracoid triangle sign is easy to assess on sagittal oblique images and, thus, is helpful for daily routine clinical work. Taken together, these data show that both thickening of the rotator interval capsule and obliteration of the subcoracoid fat triangle on MRI were useful features for diagnosing AC.

In the present MRI study, we also showed that synovitis-like abnormalities around the long biceps tendon were markedly more frequent in patients with AC compared to control subjects. Synovitis-like abnormalities at the articular surface of the subscapularis tendon or at supraspinatus tendon were not significantly different when comparing AC patients with control subjects. In contradiction with these results, Bernard et al found synovitis-like abnormalities at the superior border of the subscapularis tendon were significantly more common in patients with frozen shoulder, while synovitis-like abnormalities around the long biceps tendon were not significantly different from findings in the control group [15]. Other investigators have also shown synovitis-like abnormalities at the subscapularis tendon in patients with AC [31], [32]. Therefore, additional studies are needed to better define the role of these synovitis-like abnormalities in the diagnosis of AC.

There are two limitations in this study. On the one hand, all the patients with AC were not classified into early or late stages because of clinical staging of AC was reported to associate with synovial inflammation and capsular fibrosis [6]. On the other hand, all the clinical diagnosing patients with AC were not treated and confirmed by the arthroscopy or surgery.

Despite these limitations in our study, some results show that the MR imaging may prove valuable for assisting diagnosis of AC of shoulder joint with a highly sensitive and specific detection.
